# Comparison of Endoplasmic Reticulum Stress and Pyroptosis Induced by Pathogenic Calcium Oxalate Monohydrate and Physiologic Calcium Oxalate Dihydrate Crystals in HK-2 Cells: Insights into Kidney Stone Formation

**DOI:** 10.3390/cells13242070

**Published:** 2024-12-15

**Authors:** Wei-Jian Nong, Xin-Yi Tong, Jian-Ming Ouyang

**Affiliations:** Institute of Biomineralization and Lithiasis Research, College of Chemistry and Materials Science, Jinan University, Guangzhou 510632, China

**Keywords:** endoplasmic reticulum stress, pyroptosis, TXNIP, COM, COD

## Abstract

Endoplasmic reticulum stress (ERS) can activate pyroptosis through CHOP and TXNIP; however, the correlation between this process and the formation of kidney stones has not been reported. The purpose is to investigate the effects of calcium oxalate monohydrate (COM) and calcium oxalate dihydrate (COD) on ERS and pyroptosis in HK-2 cells and to explore the formation mechanism of calcium oxalate stones. HK-2 cells were injured by 3 μm COM and COD. COM and COD significantly upregulated the expression levels of GRP78, CHOP, TXNIP, and pyroptosis-related proteins (NLRP3, caspase-1, GSDMD-N, and IL-1β). Fluorescence colocalization revealed that COM induced pyroptosis by inducing the interaction between TXNIP and NLRP3. Both COM and COD crystals can induce ERS and pyroptosis in HK-2 cells. COM induces the interaction with NLRP3 by the upregulation of CHOP and TXNIP and then promotes pyroptosis, while COD only promotes pyroptosis by the upregulation of CHOP. The cytotoxicity and the ability of COM to promote crystal adhesion and aggregation are higher than COD, suggesting that COM is more dangerous for calcium oxalate kidney stone formation.

## 1. Introduction

Kidney stones are a common disease of the urinary system, with an incidence of about 15% [1], but the mechanism of their formation has not been elucidated [2]. More than 70% of the components in kidney stones are calcium oxalate (CaOx), mainly in the form of calcium oxalate monohydrate (COM) and calcium oxalate dihydrate (COD). The formation of CaOx stones involves crystal nucleation, growth, adhesion, and retention [3]. Studies have confirmed that renal inflammatory injury caused by crystals plays a crucial role in the occurrence and development of CaOx kidney stones [4].

Endoplasmic reticulum stress (ERS) is closely related to the activation of intracellular NOD-like receptor thermal protein domain associated protein 3 (NLRP3) inflammasome [5]. Glucose-regulated protein 78 (GRP78) is a highly expressed endoplasmic reticulum chaperone protein during ERS, so it is often used as one of ERS markers [6]. Studies [7] have found that COM can cause cellular ERS, including the activation of three pathways, namely inositol requiring enzyme 1α (IRE1α), PKR-like eukaryotic initiation factor 2α kinase (PERK), and activating transcription factor-6 (ATF6) pathway, and the upregulation of C/EBP homologous protein (CHOP). However, ERS inhibitor treatment could effectively reduce the effect of COM on cell adhesion and reduce the expression of osteopontin (OPN) and matrix gamma-carboxyglutamate (MPG), which are related to stone formation. The inhibition of ERS helps to prevent the formation of kidney stones [8,9].

Thioredoxin-interacting protein (TXNIP) is considered to be a key signal node connecting ERS and inflammation. During ERS, the high expression of the IRE1α pathway induces the activation of TXNIP, which then binds to NLRP3 and activates, leading to pro-caspase-1 cleavage and interleukin-1β (IL-1β) secretion, resulting in a series of inflammatory injuries [10,11]. TXNIP has been proved to be an ideal target for some kidney diseases, including nephrotic syndrome, acute kidney injury, and diabetic nephropathy [12,13,14,15]. However, little is known about TXNIP and kidney stone formation. Studies have found that a high expression of TXNIP occurs in the kidney of rats with hyperoxaluria and CaOx kidney stones and induces the activation of NLRP3 inflammasome, showing a strong inflammatory response [16]. Another study found that in the mouse model of ethylene glycol induced hyperoxaluria, TXNIP, NLRP3, caspase-1, and other genes were significantly upregulated when CaOx crystal deposition began in the kidney of the mice [17].

Pyroptosis is a newly discovered regulated cell death mode, which was initially considered to be dependent on caspase-1 and inflammasome-mediated cell death and has typical characteristics, such as obvious membrane perforation and inflammatory factor release [18]. NLRP3 is a sensor that detects danger signals. NLRP3 is activated by stimulation to recruit the apoptosis-associated speck-like protein containing CARD (ASC). After assembly with pro-caspase-1 into the inflammasome, pro-caspase-1 is activated and cleaved into p20 and p10 fragments. It then activates caspase-1 to cleave gasdermin-D (GSDMD) to the activated form, the N-terminal cleavage product of GSDMD (GSDMD-N), which then oligomerizes and forms membrane pores. It induces pyroptosis and releases proinflammatory cytokines IL-1β and human interleukin-18 (IL-18) [19]. Some studies suggest that the inflammatory damage induced by NLRP3 activation and its mediated pyroptosis are the nodal points for the formation of kidney stones [20,21,22]. Inhibiting CaOx crystal induced renal cell pyroptosis, including downregulating the expression of NLRP3, caspase-1, IL-1β, and other related pyroptosis proteins, can reduce the risk of kidney stone formation [23]. It has been shown [24] that the interaction between crystals and cells can lead to ERS and mitochondrial damage and promote NLRP3 activation. Several studies [25,26,27] have shown that the ERS/TXNIP/NLRP3/pyroptosis axis plays an important role in different diseases. However, whether this axis plays a role in kidney stone formation remains unknown.

In this study, HK-2 cells were damaged by COM and COD with a size of 3 μm to construct a kidney stone model in vitro, and the effects of COM and COD on ERS, TXNIP, CHOP, and pyroptosis were investigated, in order to provide enlightenment for inhibiting the formation of kidney stones and finding therapeutic targets.

## 2. Materials and Methods

### 2.1. Materials and Instruments

Materials: Human renal proximal tubular epithelial cells (HK-2) (Shanghai Cell Bank, Chinese Academy of Sciences, Shanghai, China). DMEM/F-12 medium, trypsinand bovine serum protein (BSA) were purchased from Gbico Biochemical Products LTD. Fetal Bovine Serum, FBS (Umedium, Hefei, China). Phosphate buffer solution (PBS), 4% paraformaldehyde, 4,6-diamino-2-phenylindole (DAPI) staining solution, and a CCK-8 cell proliferation detection kit were purchased from Beyotime (Shanghai, China). The human interleukin-18 ELISA kit was purchased from Beijing Solaibao Technology Co., LTD. Goat anti-mouse and goat anti-rabbit fluorescent secondary antibodies were purchased from HuaBio (Hangzhou, China). CaCl_2_, NaOx, and other chemical reagents were analytically pure and purchased from Aladdin Biochemical Technology Co., LTD. (Shanghai, China). FAM-FLICA caspase-1 Assay Kit (ImmunoChemistry Technologies LLC, Bloomington, MN, USA).

Instruments: D/max 2400 X-ray powder diffractometer (Rigaku, Tokyo, Japan); Zetasizer Nano ZS laser nanoparticle size analyzer (Mallern Company, Cheshire, UK); microplate reader (Safire2, Tecan, Männedorf, Switzerland); laser confocal microscopy (LSM510 META DUO SCAN, Zeiss, Oberkochen, Germany); fluorescence inverted microscope (Leica DMRA2, Germany); flow cytometry (Beckman, Gallios, CA, USA); ULTRA 55 field emission scanning electron microscope (Zeiss, Germany); and multifunction chemiluminescence imager (Odyssey, Lincoln, NE, USA).

### 2.2. Synthesis and Characterization of COM and COD

With reference to [28], COM and COD crystals with the size of 3 μm were synthesized and characterized by XRD, SEM, and other methods, indicating that they were the target compounds.

Zeta potential detection of COM and COD in the medium: COM and COD with a concentration of 300 μg/mL were ultrasonic for 5 min to disperse the crystals in the serum-free medium. The zeta potential of the crystals was detected by a nanometer particle size analyzer.

### 2.3. Cell Culture and Injury Model Construction

#### 2.3.1. Cell Culture

HK-2 cells were cultured in DMEM-F12 medium supplemented with 10% serum, 100 U/mL penicillin, and 100 μg/mL streptomycin at 37 °C, 5% CO_2_, and saturated humidity. Cells were digested by trypsin and passaged.

#### 2.3.2. Cell Damage

A cell suspension at a concentration of 1.0 × 10^5^ cells/mL was seeded in well plates and cultured to 80% confluence.

The experiment was divided into 3 groups:

(1) Normal control group (NC): cells were cultured in serum-free DMEM-F12 medium for 48 h;
(2) COM crystal damage group: cells were cultured in serum-free medium containing COM at the final concentration of 300 μg/mL for 48 h;
(3) COD crystal damage group: cells were cultured in serum-free medium containing COD at the final concentration of 300 μg/mL for 48 h.

### 2.4. Observation of Cytotoxicity and Crystal Adhesion of COM and COD Crystals

#### 2.4.1. Cell Viability Detected by CCK8 Assay

The experimental grouping is the same as Section 2.3.2. Parallel detection was carried out three times, and the average analysis taken. After reaching the action time, the test was carried out according to the assay method of the kit, and the OD value was detected with an enzyme marker at the wavelength of 450 nm. The formula is as follows:

cell viability (%) = [OD_(damage group)_ − OD_(blank group)_]/[OD_(control group)_ − OD_(blank group)_] (1)

#### 2.4.2. Crystal Cell Adhesion Experiment

Experiments on crystal cell adhesion were carried out with reference to [29]. Single cell suspension with a concentration of 1.0 × 10^5^ cells/mL was inoculated into the well plate and cultured for 24 h, and serum-free medium with COM and COD of 300 μg/mL was incubated for 1 h and 48 h. After the damage time was reached, the unattached crystals were washed away by strongly washing the cells with PBS five times. Use an inverted fluorescence microscope to observe and select representative images.2.5. COM and COD Crystal Induced ERS

### 2.5. COM and COD Crystal Induced ERS

#### 2.5.1. Immunofluorescence Analysis of GRP78

The experimental groups were the same as those in Section 2.3.2. The treated cells were fixed with 4% paraformaldehyde and penetrated with 0.1% TritonX-100. After blocking with blocking solution (Beyotime), the cells were incubated with GRP78 primary antibody (1:250) overnight at 4 °C, followed by incubation with goat anti-rabbit IgGH&L (HuaBio, Hangzhou, China) for 1 h at room temperature. After staining the Nuclei with DAPI, the results were observed by laser confocal microscopy. Semi-quantitative analysis was performed using ImageJ, in which the mean fluorescence intensity was calculated by dividing the corrected optical density by the total fluorescence area. The assays were performed three times in parallel.

#### 2.5.2. Western Blot Analysis for IRE1α, CHOP, ATF6, Phospho-PERK and β-Tubulin

The experimental groups were the same as those in Section 2.3.2. Total protein was extracted using RIPA lysis buffer, and protein concentrations were determined using the BCA quantification kit and adjusted to the same concentration. Proteins were separated by sodium dodecyl sulfate–polyacrylamide gel electrophoresis (SDS-PAGE). Proteins were then transferred to a nitrocellulose membrane. The resulting blots were blocked with 5% skim milk and incubated with IRE1α (1:1000; Cell Signaling Technology, Danvers, MA, USA), CHOP (1:1000; Proteintech, Wuhan, China), ATF6 (1:1000; Beyotime), Phospho-PERK (1:1000; Beyotime), and β-tubulin (1:5000; Proteintech) overnight at 4 °C. The membranes were then washed three times in PBS containing Tween-20 and incubated with anti-rabbit IgG or anti-mouse IgG secondary antibody (1:5000) for 1 h at room temperature. Proteins were detected using a chemiluminescence system. β-tubulin protein was used as a reference, and the expression of the proteins was standardized.

### 2.6. COM and COD Crystal Induced Pyroptosis

#### 2.6.1. The Level of IL-18 Was Detected by ELISA

The experimental groups were the same as those in Section 2.3.2. After reaching the time of injury, the cell culture medium supernatant was collected. After reacting with the reagent in the human interleukin-18 (IL-18) ELISA kit, the optical density (OD) value at 450 nm was measured on a microplate reader three times in parallel.

#### 2.6.2. Active Caspase-1 Detected by Flow Cytometry

The experimental groups were the same as those in Section 2.3.2. The suspended cells were digested and collected after reaching the injury time, and after incubation in the dark with FLICA-YVAD (the probe to bind with caspase-1) solution at 37 °C for 1 h, the cells were washed three times with washing buffer and resuspended in buffer containing propidium iodide (PI). Finally, each sample was analyzed for cell population by flow cytometry.

#### 2.6.3. Detection of Active Caspase-1

The experimental groups were the same as those in Section 2.3.2. FLICA-YVAD probe and PI staining were the same as in Section 2.6.2. Nuclei were stained by adding Hoechst 33,342 solution and incubated at 37 °C for 6 min. They were washed three times with 1 mL PBS solution. The expression of caspase-1 and nuclear staining were observed under laser confocal fluorescence microscope.

#### 2.6.4. Western Blot Analysis for NLRP3, Pro-Caspase-1, GSDMD-N, IL-1β and β-Tubulin

The experimental grouping was the same as Section 2.3.2, and the Western blot analysis method was the same as Section 2.5.2. NLRP3 (1:5000; Proteintech), pro-caspase-1 (1:2500; Proteintech), GSDMD-N (1:1000; Cell Signaling Technology), and IL-1β (1:1000; Proteintech), using β-tubulin protein as a reference and normalizing protein expression.

### 2.7. Interaction Between TXNIP and NLRP3

#### 2.7.1. Western Blot Analysis for TXNIP

The experimental grouping was the same as Section 2.3.2, and the Western blot analysis method was the same as Section 2.5.2. The primary antibody was prepared with TXNIP (1:1000; Proteintech).

#### 2.7.2. NLRP3 Colocalizes with TXNIP

The experimental grouping was the same as Section 2.3.2. The cells were fixed with 4% paraformaldehyde and then treated with NLRP3 (1:250, Proteintech) and TXNIP (1:250; Proteintech) and incubated overnight at 4 °C. After incubation with secondary antibodies mixed goat anti-mouse IgGH&L (Huabio, Hangzhou, China) and goat anti-rabbit IgGH&L (Huabio, Hangzhou, China) for 1 h at room temperature, nuclei were counterstained with DAPI. The results were observed by laser confocal microscopy.

### 2.8. Statistical Analysis

The normal distribution of experimental results was analyzed by the Shapiro–Wilk test. Data were assessed using one-way ANOVA test, followed by Tukey’s multiple comparison test for those following normal distribution. The data were presented as individual values and assessed using the Kruskal–Wallis test, followed by Dunn’s multiple comparisons test when following a nonnormal distribution. Statistical analyses were performed with Prism 6.0 (GraphPad Software, San Diego, CA, USA).

## 3. Results

### 3.1. Synthesis and Characterization of COM and COD

**Figure 1:**
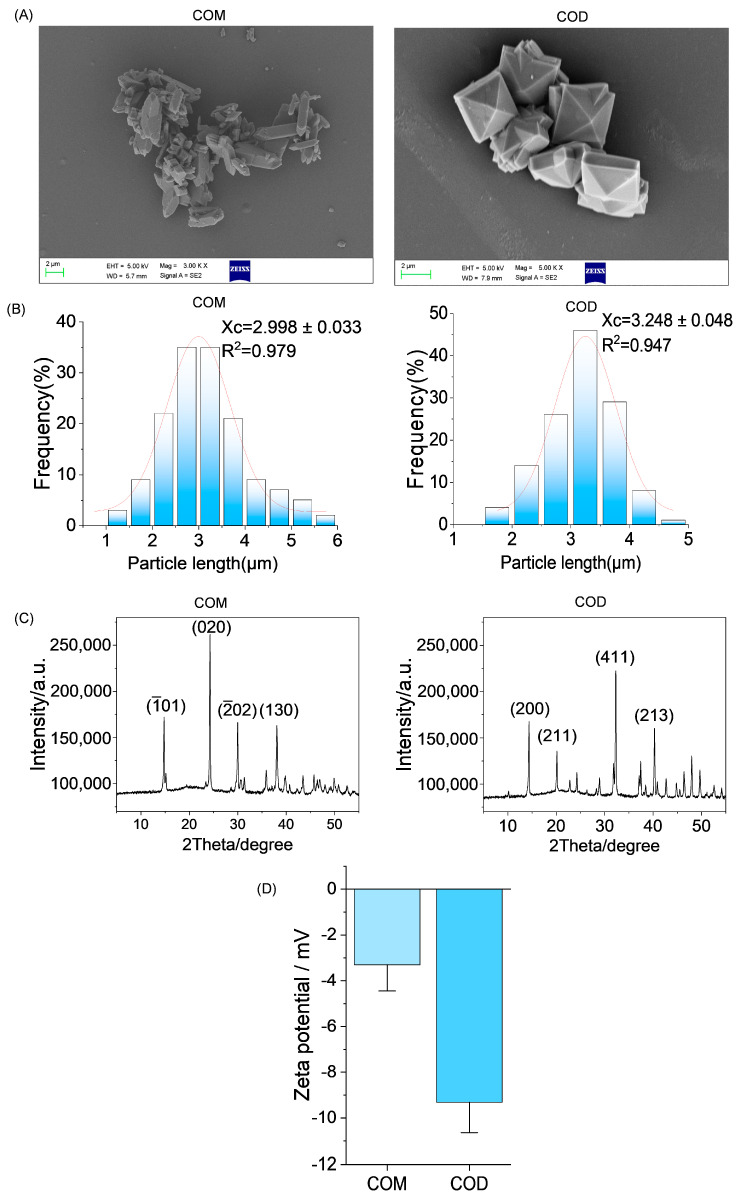
Synthesis and characterization of COM and COD. (**A**) SEM; (**B**) the particle size distributions fitted to normal distribution curves (The red curve is a normal fitting distribution); (**C**) crystal XRD pattern; (**D**) zeta potential. Calcium oxalate monohydrate, COM. Calcium oxalate dihydrate, COD. Scanning electron microscope, SEM. X-ray diffraction, XRD. Data were extracted from independent samples, and experiments were performed in triplicate.

Figure 1A,B shows SEM images of COM and COD synthesized by reference [28] and the size of crystals measured by a nano measure. The average size of COM and COD was close to 3 μm (2.998 μm and 3.248 μm, respectively). COM is an elongated hexagon with some forked crystal shapes. The COD is mainly quadrangular biconical.

Figure 1C shows the XRD patterns of COM and COD. The diffraction peaks of COM at 2θ = 14.88°, 24.2°, 30.16°, and 38.24° belonged to ($\bar{1}$01), (020), ($\bar{2}$02), and (130) crystal planes, respectively (PDF card number: 20-231). The diffraction peaks of COD were detected at 2θ = 14.36°, 20.12°, 32.24°, and 40.26°, which belonged to the (200), (211), (411), and (213) sides of COD crystal, respectively (PDF card number: 20-0233). The XRD patterns of COM and COD showed no miscellaneous peaks, indicating that the synthesized COM and COD were pure phase crystals.

Zeta potential (ζ) is a parameter that indicates the strength of mutual exclusion between particles. When the absolute value of the zeta potential (ζ) is higher, the electrostatic repulsion between particles is higher, the aggregation is less easy, and the stability is higher. Figure 1D shows that the absolute value of Zeta potential of COM (−3.3 ± 1.1 mV) was smaller than that of COD (−9.3 ± 1.3 mV) in cell culture medium, indicating that COM is easier to aggregate than COD.

### 3.2. Cytotoxicity and Cell Adhesion of COM and COD

**Figure 2:**
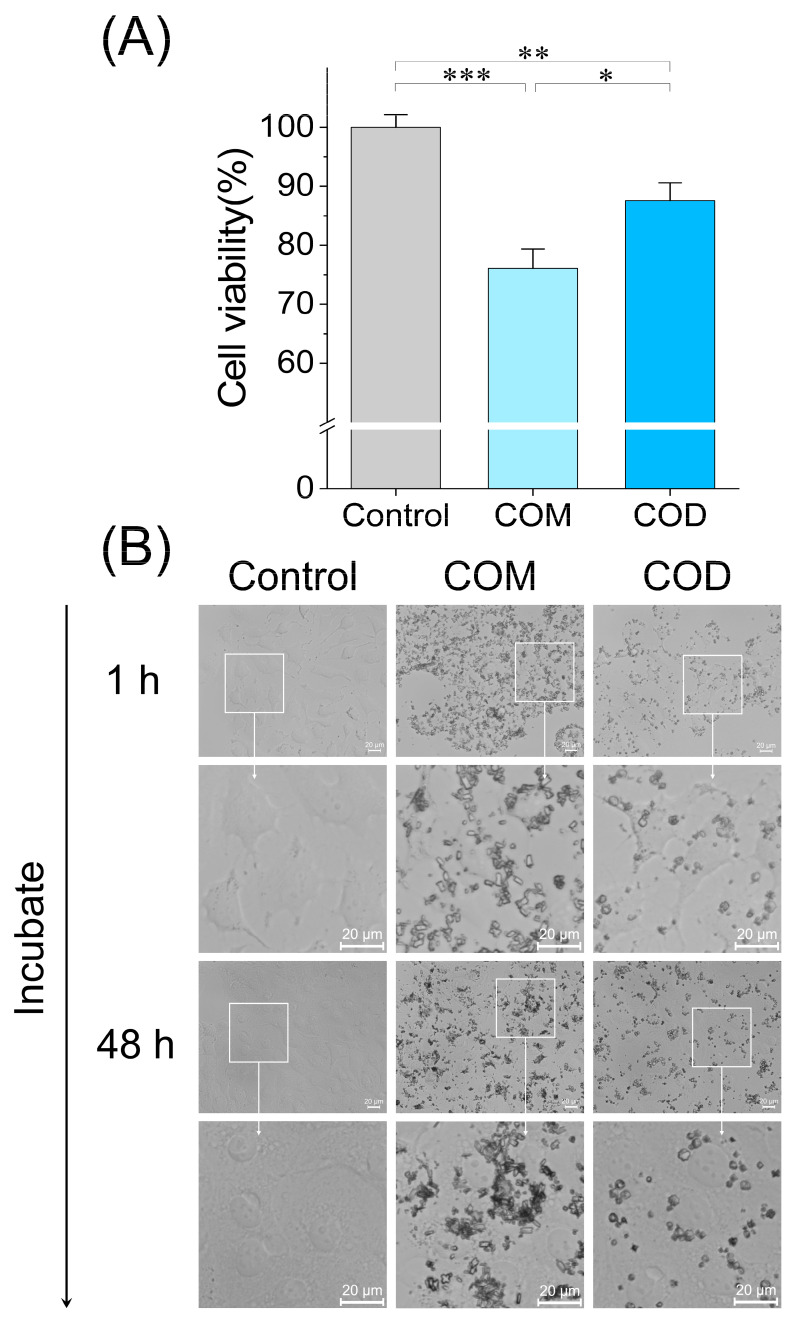
Cytotoxicity of COM and COD and their differences in adhesion to HK-2 cells. (**A**) Cell viability was measured by CCK8; (**B**) microscope images of crystal adhesion after 1 h and 48 h exposure to HK-2 cells. Control: normal control group; COM: 3 μm COM with a concentration of 300 μg/mL; COD: 3 μm COD with a concentration of 300 μg/mL; comparison among different groups, * *p* < 0.05; ** *p* < 0.01; *** *p* < 0.001. Scale bar: 20 μm. Calcium oxalate monohydrate, COM. Calcium oxalate dihydrate, COD. Data were extracted from independent samples, and experiments were performed in triplicate. The white box is the enlarged area, and the images pointed by the arrow is the enlarged images in the white box area.

The assessment of cell viability is one of the means to explore the cytotoxicity of crystals. The viability of HK-2 cells injured by COM and COD decreased (Figure 2A), and the cell viability of the COM group (76.1%) was lower than that of the COD group (87.6%), indicating that COM was more cytotoxic than COD.

Crystal aggregation and adhesion are the key processes of stone formation [3]. Figure 2B visually shows the case of crystal adhesion. After 1 h of incubation, COM and COD adhered to the cell surface to varying degrees, and the number of adhered crystals in the COM group was much higher than that in the COD group. After continued incubation until 48 h, the crystals in the COM group were significantly aggregated. In contrast, no significant aggregation of the COD crystals was observed. Figure 2B indicates that both the adhesion and aggregation of COM to HK-2 cells significantly exceeded that of COD.

### 3.3. Detection of ERS in HK-2 Cells Induced by COM and COD

**Figure 3:**
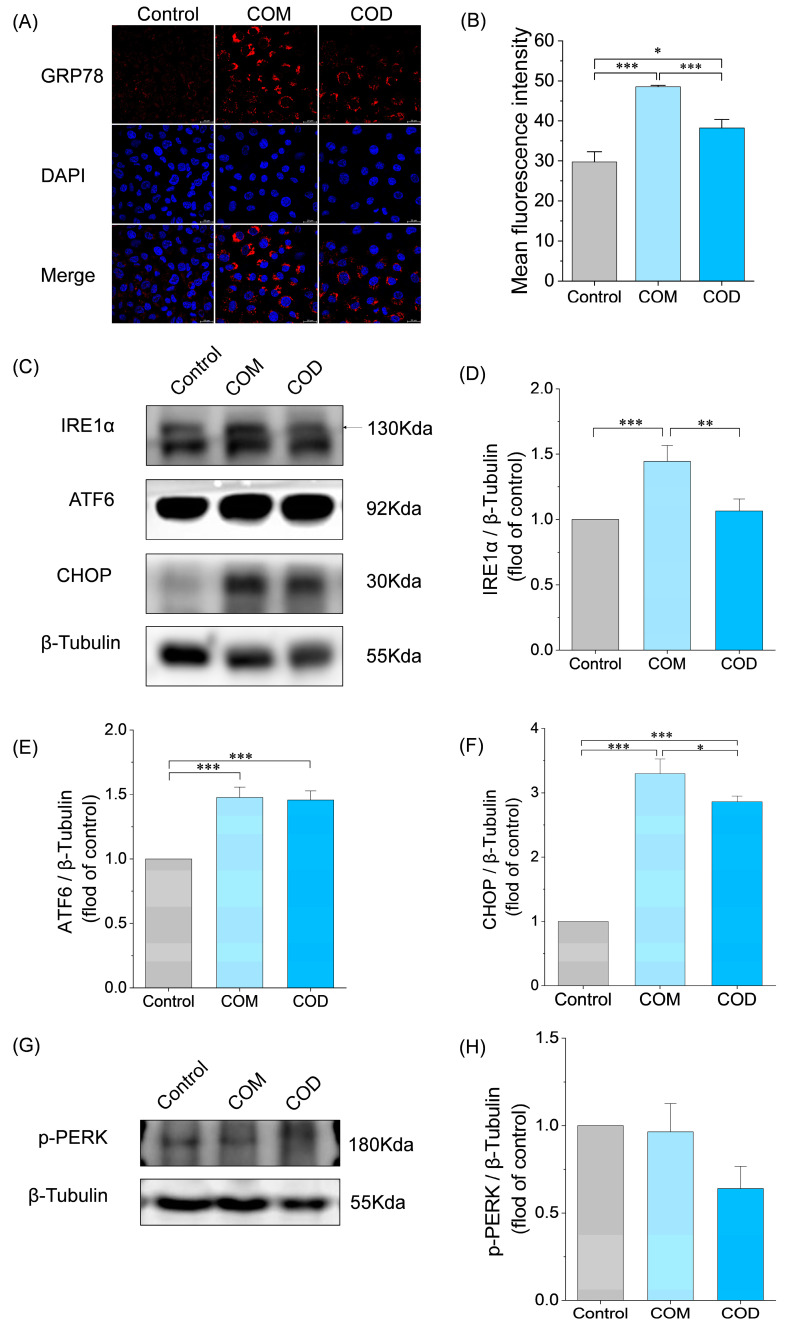
ERS induced by COM and COD. (**A**) The expression of GRP78 was observed by immunofluorescence (scale: 20 μm); (**B**) semi-quantitative analysis of GRP78 fluorescence images; (**C**,**G**) Western blot analysis of endoplasmic reticulum stress-related proteins; (**D**–**F**,**H**) semi-quantitative analysis histograms of IRE1α, ATF6, CHOP, and P-PERK, respectively. Control: normal control group; COM: 3 μm COM with a concentration of 300 μg/mL; COD: 3 μm COD with a concentration of 300 μg/mL; comparison among different groups, * *p* < 0.05; ** *p* < 0.01; and *** *p* < 0.001. Calcium oxalate monohydrate, COM. Calcium oxalate dihydrate, COD. Glucose-regulated protein 78, GRP78. 4,6-diamino-2-phenylindole, DAPI. Inositol requiring enzyme 1α, IRE1α. Activating transcription factor-6, ATF6. C/EBP homologous protein, CHOP. Phosphorylated PERK, p-PERK. Data were extracted from independent samples, and experiments were performed in triplicate.

Some studies have shown that COM causes ERS after interacting with cells, leading to the occurrence of inflammation and damage to cells [30], but no report on the effect of COD on ERS has been found. Immunofluorescence (Figure 3A,B) and Western blot (Figure 3C,F) showed that COM and COD significantly upregulated the expression of ERS chaperone GRP78 and downstream protein CHOP, while COM induced a higher expression of GRP78 and CHOP than COD, indicating that both COM and COD could activate ERS. Moreover, the induction effect of COM was higher than that of COD.

To explore which pathway was involved in COM- and COD-induced ERS, we detected the expression of phosphorylated PERK (p-PERK), IRE1α, and ATF6 by Western blot (Figure 3C–H). COM significantly increased the expression of IRE1α and ATF6, while COD only increased the expression of ATF6. Both of them had no significant effect on the expression of p-PERK. This may indicate that COM can mediate ERS through more pathways and cause more intense stress damage than COD.

### 3.4. COM- and COD-Induced Pyroptosis

Intracellular caspase-1 can be activated by the stimulated NLRP3 inflammasome and assembled into protein complexes, which cleave GSDMD to form holes in the cell membrane and release inflammatory factors, such as IL-1β, leading to the occurrence of pyroptosis [31]. The FAM-FLICA probe is a non-cytotoxic fluorescent probe that binds to the activated caspase-1 enzyme in cells with high intensity and shows green fluorescence.

**Figure 4:**
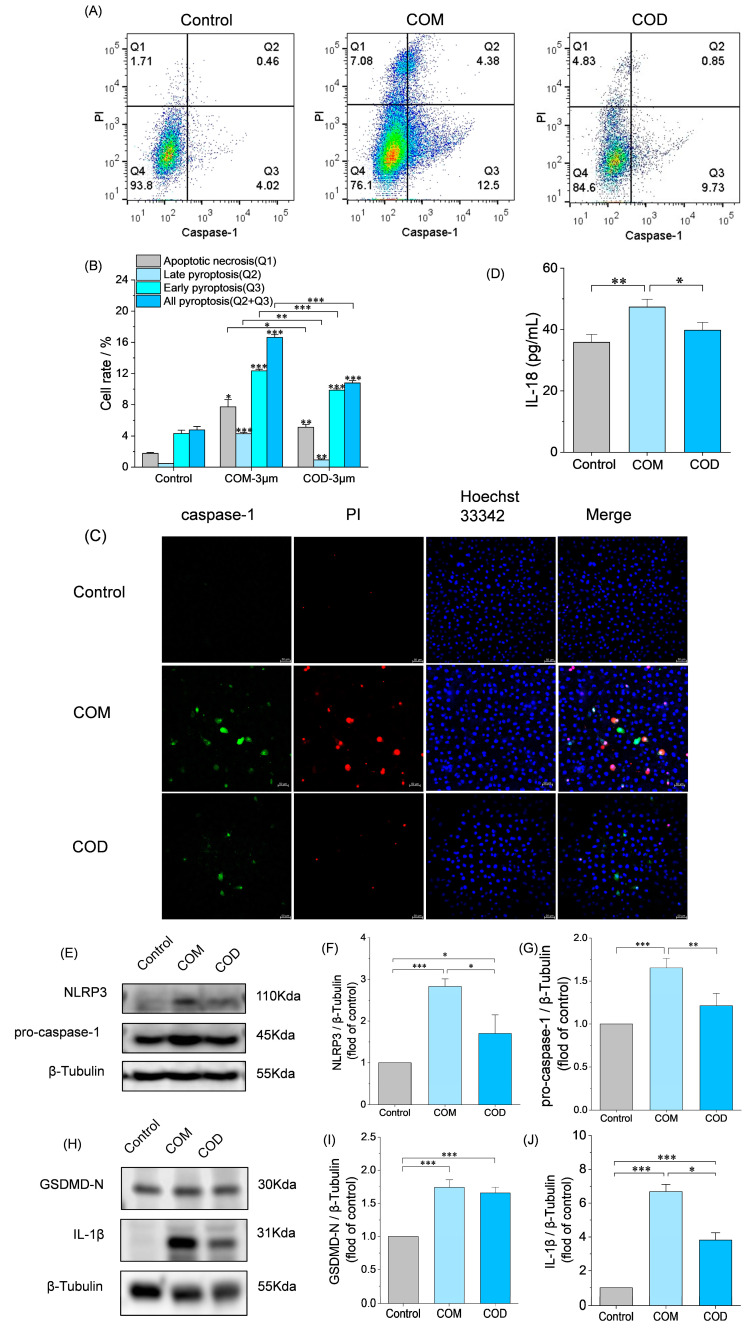
COM- and COD-induced pyroptosis and their differences. (**A**) Double staining flow quantitative analysis of caspase-1/PI; (**B**) quantitative statistical histogram of pyroptosis; (**C**) caspase-1/PI double dye confocal observation, scale: 50 μm; (**D**) semi-quantitative analysis of IL-18 in supernatant after cell injury by Elisa. (**E**,**H**) Western blot analysis of pyroptosis related pathway proteins. (**F**,**G**,**I**,**J**) semi-quantitative histograms of NLRP3, pro-caspase-1, GSDMD-N, and Pro-IL-1β, respectively. Control: normal control group; COM: 3 μm COM with a concentration of 300 μg/mL; COD: 3 μm COD with a concentration of 300 μg/mL; comparison among different groups, * *p* < 0.05; ** *p* < 0.01; *** *p* < 0.001. Calcium oxalate monohydrate, COM. Calcium oxalate dihydrate, COD. Propidium iodide, PI. N-terminal cleavage product of GSDMD, GSDMD-N. Interleukin-1β, IL-1β. NOD-like receptor thermal protein domain associated protein 3, NLRP3. Data were extracted from independent samples, and experiments were performed in triplicate. The FLICA-YVAD probe binds to caspase-1 and is excited as green fluorescence. PI binds to the nuclei of the cells with membrane rupture and was excited as red fluorescence. DAPI bound to the nuclei of all cells and was excited as blue fluorescence. More intense green and red fluorescence represents more intense pyroptosis.

To explore the role of pyroptosis in the formation of kidney stones, the expression of activated caspase-1 was detected by caspase-1/PI double staining. The proportion of activation of caspase-1 in Figure 4A is (Q2 + Q3), and it can be seen that COM (16.88%) > COD (10.58%) > normal group (4.48%).

At the same time, the pyroptosis was observed more intuitively by caspase-1/PI/Hoechst 33,342 triple staining (Figure 4C). The greener the fluorescence, the stronger the activity of caspase-1. A higher amount of red fluorescence indicates a higher amount of cell death. Figure 4C shows that the amount and intensity of red and green fluorescence in the COM group were significantly higher than those in COD group, indicating that the degree of pyroptosis induced by COM was higher than that of COD, which was basically consistent with the trend of the caspase-1/PI double staining method.

The expression of relevant pyroptotic proteins and inflammatory factors was measured with the use of Western blot and Elisa (Figure 4D–J). COM significantly upregulated the expression of NLRP3, GSDMD-N, pro-caspase-1, IL-1β, and IL-18. In contrast, although COD significantly upregulated the expression of NLRP3, GSDMD-N, and IL-1β, the expression of pro-caspase-1 and IL-18 was not significantly upregulated. The expression of COD group was lower than that of COM group. This suggests that both COM and COD can mediate pyroptosis in HK-2 cells through the classical pathway NLRP3/caspase-1/IL-1β, and COM induces a higher degree of pyroptosis than COD.

### 3.5. COM Causes Pyroptosis by Inducing TXNIP Activation and Interacting with NLRP3

A large number of studies have shown that the multifunctional protein TXNIP is a key bridge connecting ERS and the activation of NLRP3 inflammasome. The activation of the three ERS pathways (IRE1α, ATF6, and PERK) generally increased the activities of the downstream proteins CHOP and TXNIP. TXNIP activation can induce the activation of the NLRP3 inflammasome, which eventually activates caspase-1, stimulates the maturation and release of IL-1β and IL-18, and causes pyroptosis [15,32].

**Figure 5:**
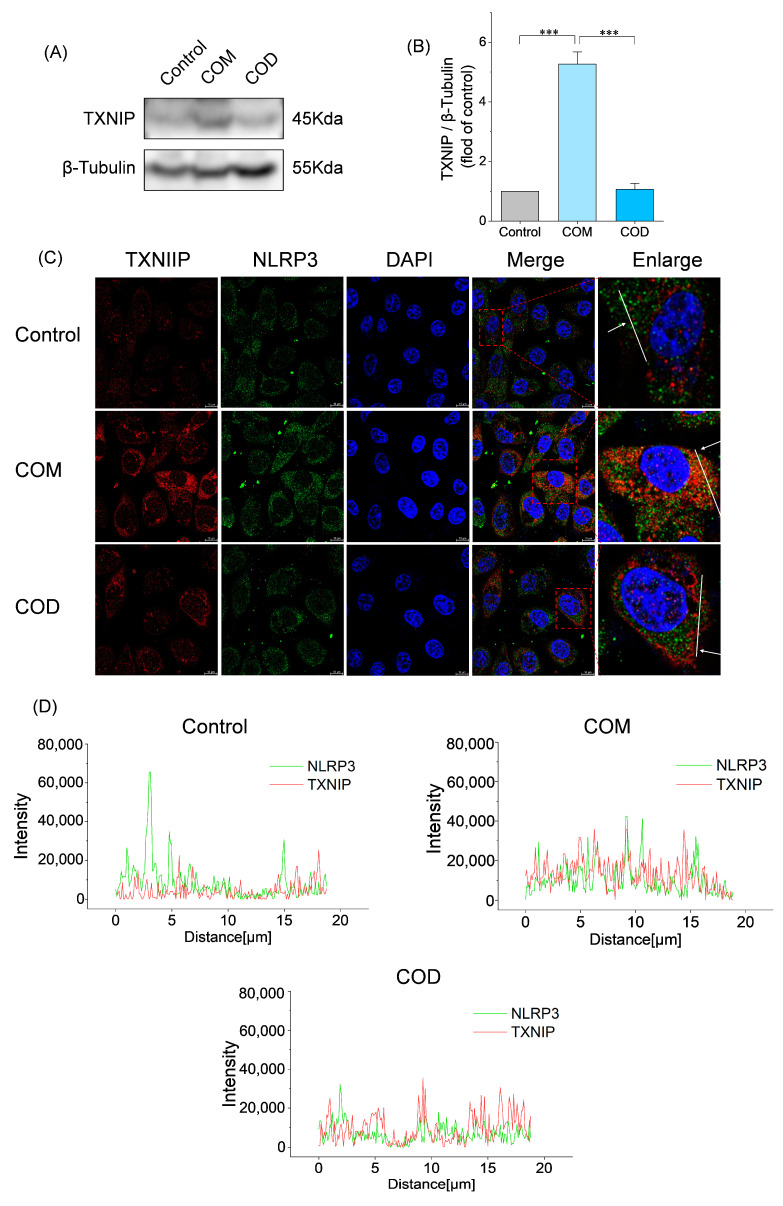
Activation effects of COM and COD on TXNIP. (**A**) Western blot analysis of TXNIP; (**B**) semi-quantitative analysis histogram of TXNIP; (**C**) visualization of the colocalization of NLRP3 and TXNIP in HK-2 cells by laser confocal microscopy, scale: 10 μm; (**D**) copositioning curve analysis diagram for the white line region of figure (**C**). Control: normal control group; COM: 3 μm COM with a concentration of 300 μg/mL; COD: 3 μm COD with a concentration of 300 μg/mL; comparison among different groups, *** *p* < 0.001. Calcium oxalate monohydrate, COM. Calcium oxalate dihydrate, COD. Thioredoxin-interacting protein, TXNIP. NOD-like receptor thermal protein domain associated protein 3, NLRP3. Data were extracted from independent samples, and experiments were performed in triplicate. TXNIP is observed as red fluorescence. NLRP3 is observed as green fluorescence. DAPI binding nuclei is observed as blue fluorescence. The image on the far right is a magnified view of the red box.

To explore the role of TXNIP in kidney stone formation, the effects of COM and COD on TXNIP were explored by Western blot (Figure 5A). It can be seen that COM induced a significant upregulation of TXNIP expression, while COD did not change significantly. Fluorescence colocalization revealed the interaction between TXNIP and NLRP3 (Figure 5C). It can be seen that the fluorescence intensity of NLRP3 (green) and TXNIP (red) in the COM group was strong, and there was obvious fluorescence colocalization. However, the intensity of TXNIP (red) in the COD group was weak, and the fluorescence colocalization of the two was not obvious. The colocalization curve analysis of the white line region (Figure 5D) showed that the colocalization curve of COM had a high degree of overlap between the NLRP3 (green) and TXNIP (red) curves, which further verified that COM induced the interaction between TXNIP and NLRP3. These results suggest that COM, but not COD, can further mediate NLRP3 activation by inducing the interaction between TXNIP and NLRP3.

## 4. Discussion

### 4.1. Differences in Cytotoxicity and Adhesion Between COM and COD

COM and COD are the main components of CaOx stones. Studies have shown that COM and COD can damage kidney cells to different degrees or in different forms and promote crystal adhesion and retention, eventually leading to the formation of kidney stones [33]. However, the mechanism of CaOx stone formation has not been fully elucidated.

We synthesized COM and COD crystals with sizes close to 3 μm (Figure 1). The ($\bar{1}$01) crystal face of COM crystal is large and rich in Ca^2+^ ions (density 0.0542 sites/ Å^2^), which has a strong binding ability to the cell surface [34,35]. In addition, COM crystals have sharp edges (Figure 1A), which also cause irregular damage to the cell membrane and local strong physical stress [36], so the interaction between COM and HK-2 cells has stronger COD, that is, the cytotoxicity of COM is greater (Figure 2A).

After incubation at the same crystal concentration for 1 h and 48 h, the adhesion ability of COM to HK-2 cells was much higher than that of COD (Figure 2B), which is consistent with the recognition that COM has a greater binding ability to cells than COD [37]. Our previous results showed that treatment with microscale COM and COD crystals adhered to and damaged the cell membrane of Vero cells and induced an increase in ROS levels. COM crystals cause more severe damage to Vero cells than COD crystals of the same size, because the adhesion ability of COM crystals is greater than that of COD crystals [38,39].

The regrowth and aggregation of crystals after adhesion to renal tubular cells is an important mechanism of kidney stone formation [40]. We observed that, as the interaction time between crystals and cells increased from 1 h to 48 h, the adhesion and aggregation of COM and COD were further aggravated (Figure 2B), indicating that the damaged HK-2 cells promoted the re-adhesion and retention of crystals. The aggregation of COM is obviously more serious than that of COD. One reason is that the absolute value of the zeta potential of COM is smaller than that of COD (Figure 1D). The smaller the absolute value of the zeta potential, the smaller the electrostatic repulsion between crystals, and the easier it is to aggregate [41].

### 4.2. Difference in ERS Induced by COM and COD

Endoplasmic reticulum is an important subcellular organelle involved in cellular metabolism. When there are high levels of misfolded proteins in the endoplasmic reticulum, it is called ERS. The formation of kidney stones is closely related to cell damage, and ERS is one of the factors that promote cell damage. CaOx crystals induce apoptosis and inflammation by mediating ERS. However, reducing CaOx-induced ERS can effectively reduce crystal adhesion and apoptosis, thereby alleviating cell damage [42,43].

Although there are many studies on COM and ERS, COD and ERS have not been reported. In this paper, it was found that both COM and COD exposure to HK-2 cells significantly upregulated the expression of GRP78 and CHOP (Figure 3A,C), indicating that both COM and COD could induce ERS to a certain extent, and COM was more effective than COD.

Studies have shown that [7] COM mediates ERS by upregulating GRP78, activating IRE1α and ATF6, and phosphorylating PERK, causing the upregulation of CHOP activity and then increasing the adhesion of crystals to cells. Interestingly, phosphorylated PERK was upregulated in the first 6 h of COM exposure in HK-2 cells, and then its expression level continued to decrease until 48 h, even lower than that of the control group [7]. This is consistent with our experimental results (Figure 3G), which showed that COM and COD had no significant effect on p-PERK after 48 h. COM significantly upregulated the expression of IRE1α and ATF6, while COD only significantly upregulated the expression of ATF6 and had no significant effect on IRE1α (Figure 3C). In the ERS, the activation of three pathways, IRE1α, PERK, and ATF6, will promote CHOP activity [44], and the higher expression of CHOP induced by COM than COD (Figure 3C,F) can be attributed to COM promoting CHOP activity through multiple pathways (Figure 3C). In conclusion, COM is more capable of inducing ERS and damaging cells than COD.

### 4.3. Differences in Pyroptosis Induced by COM and COD

A continuously high level of ERS is the key to causing pyroptosis [45,46,47], because CHOP expressed in large amounts in ERS is one of the important causes of pyroptosis [48,49], evidenced by the use of siRNA to silence the expression of CHOP, which can effectively reduce the activation of NLRP3 [50]. The classical pyroptosis is a caspase-1-dependent programmed cell death, involving the assembly of NLRP3 inflammasome and the cleavage of GSDMD and promoting the release of inflammatory factors IL-18 and IL-1β [51]. CaOx crystals can induce various types of cell death, such as apoptosis and necrosis [24].

Studies have shown that COM crystals induce NLRP3 activation and lead to pyroptosis, which is considered to be one of the causes of kidney stone formation [52]. Ding et al. [23] confirmed that the levels of GSDMD, NLRP3, cleaved caspase-1, and mature IL-1β were increased in kidney stone mice. Com-treated HK-2 cells showed upregulated GSDMD-N and increased lactate dehydrogenase (LDH) release. Similarly, COM can significantly upregulate the expression of NLRP3, pro-caspase-1, GSDMD-N, IL-18, and IL-1β (Figure 4D–H), indicating that COM induces pyroptosis in HK-2 cells through the classical pathway.

However, the association of COD crystals, as well as CaOx crystals, with pyroptosis has not been reported. In this study, COD significantly upregulated the expression of NLRP3, GSDMD-N, and IL-1β, and the upregulation was generally lower than that of COM. In this study, the proportion of COM- and COD-induced activation of caspase-1 was detected by caspase-1/PI double staining (Figure 4A). The activation of caspase-1 was COM (16.88%) > 3 μm COD (10.58%) (Figure 4B). This trend was confirmed by caspase-1/PI/Hoechst 33,342 triple staining (Figure 4C). It can be seen that COM injury induced greater pyroptosis in HK-2 cells compared with COD. Therefore, we conclude that COD is also capable of inducing pyroptosis, but less so than COM. The difference in pyroptosis induced by COM and COD may be attributed to the difference in crystal–cell interaction, namely crystal adhesion, and the difference in cytotoxicity. Crystal adhesion to cells can lead to pyroptosis. Compared with COD crystals, COM crystals adhere to the cell membrane in larger numbers (Figure 2B), have a greater activation of caspase-1 (Figure 4A), and the expression of proteins in the pyroptosis signaling pathway is also significantly upregulated (Figure 4D–J). Our study has deepened the understanding of the difference between pathological COM crystals and physiological COD crystals [35]. The data provided support the benefit of reducing the incidence of kidney stones by inducing the conversion of COM to COD.

### 4.4. TXNIP May Be a Potential Target to Mediate the Central Signal of Pyroptosis and Inhibit Stone Formation After COM-Induced ERS

TXNIP is a multifunctional protein, which plays an important role in oxidative stress, ERS, and inducing inflammation, and has been widely studied in diabetes and other chronic kidney diseases [53]. TXNIP is considered a key protein in cellular metabolism and its stress response pathway and a potential therapeutic target [15]. Previous studies have shown that ERS mediates TXNIP activation and interacts directly with NLRP3, leading to the assembly of the NLRP3 inflammasome complex, which eventually activates caspase-1 and stimulates the maturation and release of IL-1β and IL-18, leading to the generation of pyroptosis [9,32]. However, there are few reports on the role of TXNIP in kidney stone disease. Only studies show that TXNIP, NLRP3, and other genes are upregulated in the in vivo model of kidney stone mice [17], but they do not link ERS with pyroptosis.

**Figure 6:**
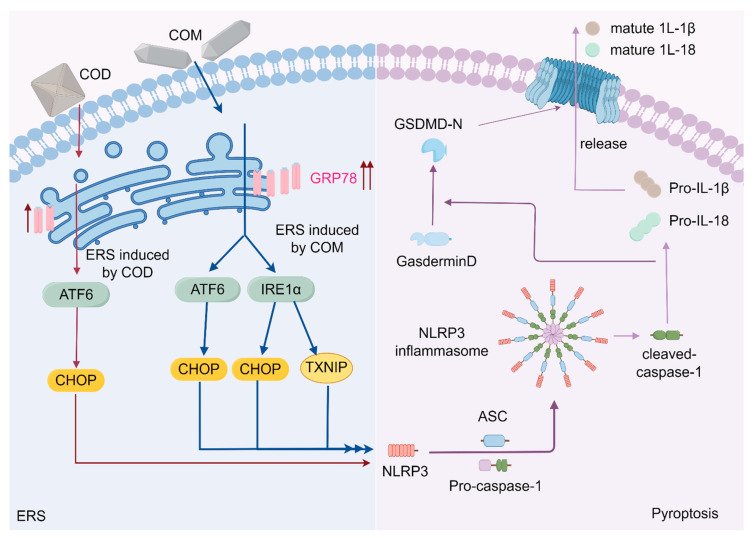
The mechanism of COM and COD damages HK-2 cells through the ERS–NLRP3 pyroptosis pathway and promotes the formation of kidney stones (by Figdraw). Calcium oxalate monohydrate, COM. Calcium oxalate dihydrate, COD. Glucose-regulated protein 78, GRP78. Endoplasmic reticulum stress, ERS. Activating transcription factor-6, ATF6. Inositol requiring enzyme 1α, IRE1α. C/EBP homologous protein, CHOP. Thioredoxin-interacting protein, TXNIP. NOD-like receptor thermal protein domain associated protein 3, NLRP3. N-terminal cleavage product of GSDMD, GSDMD-N. Interleukin-18, IL-18. Interleukin-1β, IL-1β. Arrows indicate activation or upregulation effects.

Previous studies have shown that the high expression of IRE1α during ERS can mediate the activation of TXNIP and further promote the assembly of the inflammasome NLRP3 [10,11]. This is consistent with our finding that COM induces a high expression of IRE1α (Figure 3C,D) and mediates TXNIP activation; whereas, COD does not activate IRE1α and TXNIP. Immunofluorescence colocalization revealed that COM mediated the interaction between TXNIP (red) and NLRP3 (green) (Figure 5C), suggesting that COM may induce the activation of TXNIP and interact with NLRP3 to cause the assembly of NLRP3 inflammasome, leading to pyroptosis (Figure 6).

As mentioned above, ERS activates CHOP and TXNIP, both of which can interact with NLRP3 to induce pyroptosis; that is, there are ERS-CHOP pyroptosis and ERS-TXNIP pyroptosis processes. COD crystal-induced pyroptosis was solely attributed to the ERS-CHOP pyroptosis process described above. COM crystals not only induced increased CHOP expression to a greater extent but also increased TXNIP expression, and ERS-CHOP pyroptosis and ERS-TXNIP pyroptosis occurred simultaneously (Figure 6). Thus, COM crystals induced stronger ERS, more expression of CHOP and TXNIP, and greater pyroptosis compared with COD crystals.

Pyroptosis is involved in the formation of kidney stones. Chen et al. [54] demonstrated that GSDMD is involved in calcium oxalate genesis and kidney stone progression through in vitro and in vivo experiments. Ding et al. [23] initially verified that pyroptosis mediates kidney stones, with elevated levels of GSDMD as well as activated NLRP3, cleaved caspase-1, and mature IL-1β in mice with kidney stones. COM-treated renal tubular epithelial cell models showed that COM increased cleaved GSDMD protein levels and cellular LDH release [23].

Our study indicated that COM crystals promote pyroptosis through multiple pathways in ERS (Figure 6) by adhering to the cell membrane, damaging cells, and releasing the proinflammatory factors IL-1β and IL-18. Such an inflammatory environment may further promote crystal adhesion and aggregation [55], promote the biomeramalization process involving collagen and osteopontin [55], and eventually, lead to the formation of kidney stones.

Our study highlights the difference in pyroptosis induced by COM and COD crystals: COM crystals were able to upregulate TXNIP, but COD crystals were not (Figure 5A). Therefore, TXNIP may be a potential target for mediating the central signal of pyroptosis and inhibiting stone formation after COM-induced ERS. There are many kinds of cells in stone study, including renal tubular cells (proximal, distal, collecting duct), renal interstitial fibroblasts, macrophages, and so on. This paper has some limitations, such as the single cell line and the need for clinical verification. The current findings are derived from in vitro study, and further in vivo and animal experiments are needed to corroborate the present findings.

## 5. Conclusions

Here, we propose a mechanism of renal cell injury in which COM leads to pyroptosis via the ERS-TXNIP-NLRP3 pathway. The early adhesion of COM and COD crystals to cells and their interaction with HK-2 cells can induce pyroptosis by mediating ERS, leading to cell damage, further accelerating crystal adhesion and aggregation, and finally, promoting the formation of kidney stones. On the other hand, COD did not have the ability to regulate TXNIP, and the effect of inducing ERS and pyroptosis was weak. COD-induced pyroptosis was attributed to COD, causing ERS and mediating CHOP activation, indicating the weak damage of COD on renal cells. Taken together, our study suggests that TXNIP is a promising target for inhibiting renal stone formation. This study also validates the promotion of COM conversion to COD as a potential approach to reduce renal cell damage.

## Data Availability

The original contributions presented in this study are included in the article. Further inquiries can be directed to the corresponding author.
